# Supplementary material for: Droplet-based bisulfite sequencing for high-throughput profiling of single-cell DNA methylomes
Source: Nat Commun. 2023 Aug 3;14:4672. doi: 10.1038/s41467-023-40411-w (PMC10400590; doi:10.1038/s41467-023-40411-w)
Supplement: Supplementary file 1 — Supplementary Information [file 41467_2023_40411_MOESM1_ESM.pdf]

Supplementary Information for:

Droplet-based bisulfite sequencing for high-throughput profiling of single-cell DNA methylomes

Qiang Zhang[1], Sai Ma[2,5], Zhengzhi Liu[2], Bohan Zhu[1], Zirui Zhou[1], Gaoshan Li[1], J. Javier Meana[3], Javier González-Maeso[4], Chang Lu[1]

[1] Department of Chemical Engineering, Virginia Tech, Blacksburg, VA 24061, USA.

[2] Department of Biomedical Engineering and Mechanics, Virginia Tech, Blacksburg, VA 24061, USA.

[3] Department of Pharmacology, University of the Basque Country UPV/EHU, CIBERSAM, Biocruces Health Research Institute, E-48940 Leioa, Bizkaia, Spain.

[4] Department of Physiology and Biophysics, Virginia Commonwealth University School of Medicine, Richmond, VA 23298, USA.

[5] Present address: Department of Genetics and Genomic Sciences, Icahn School of Medicine at Mount Sinai, New York, NY 10029, USA.

Correspondence should be addressed to C.L. (email: changlu@vt.edu).

Supplementary Figures

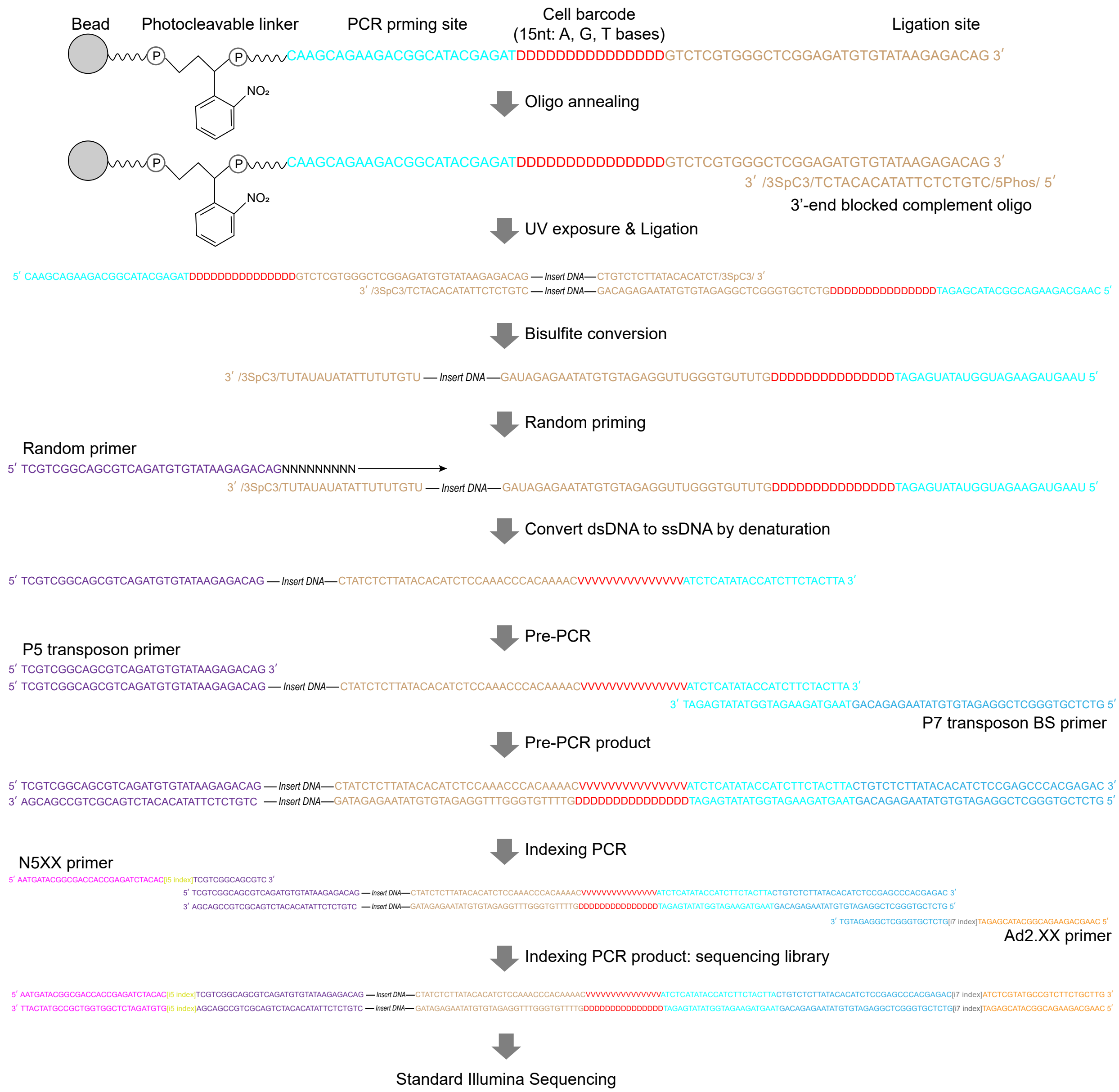

Supplementary Figure 1 Oligonucleotide and primer sequences involved in the construction of Drop-BS library.

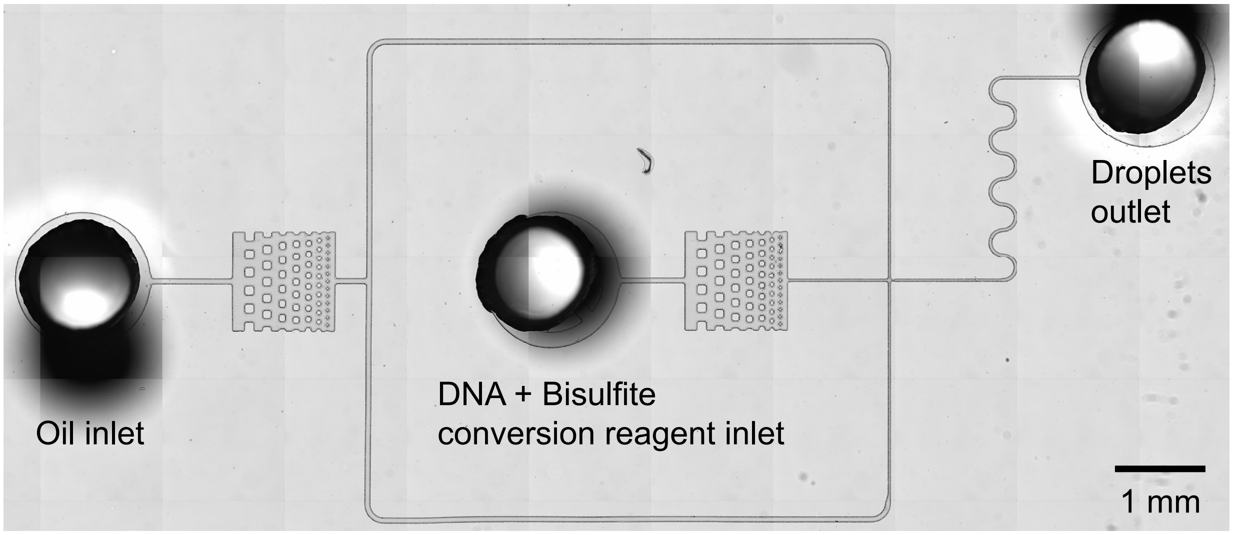

Supplementary Figure 2 The bisulfite droplet device used in Drop-BS. Filtration structures were placed between the inlets and the narrow channels to trap particles and prevent clogging.

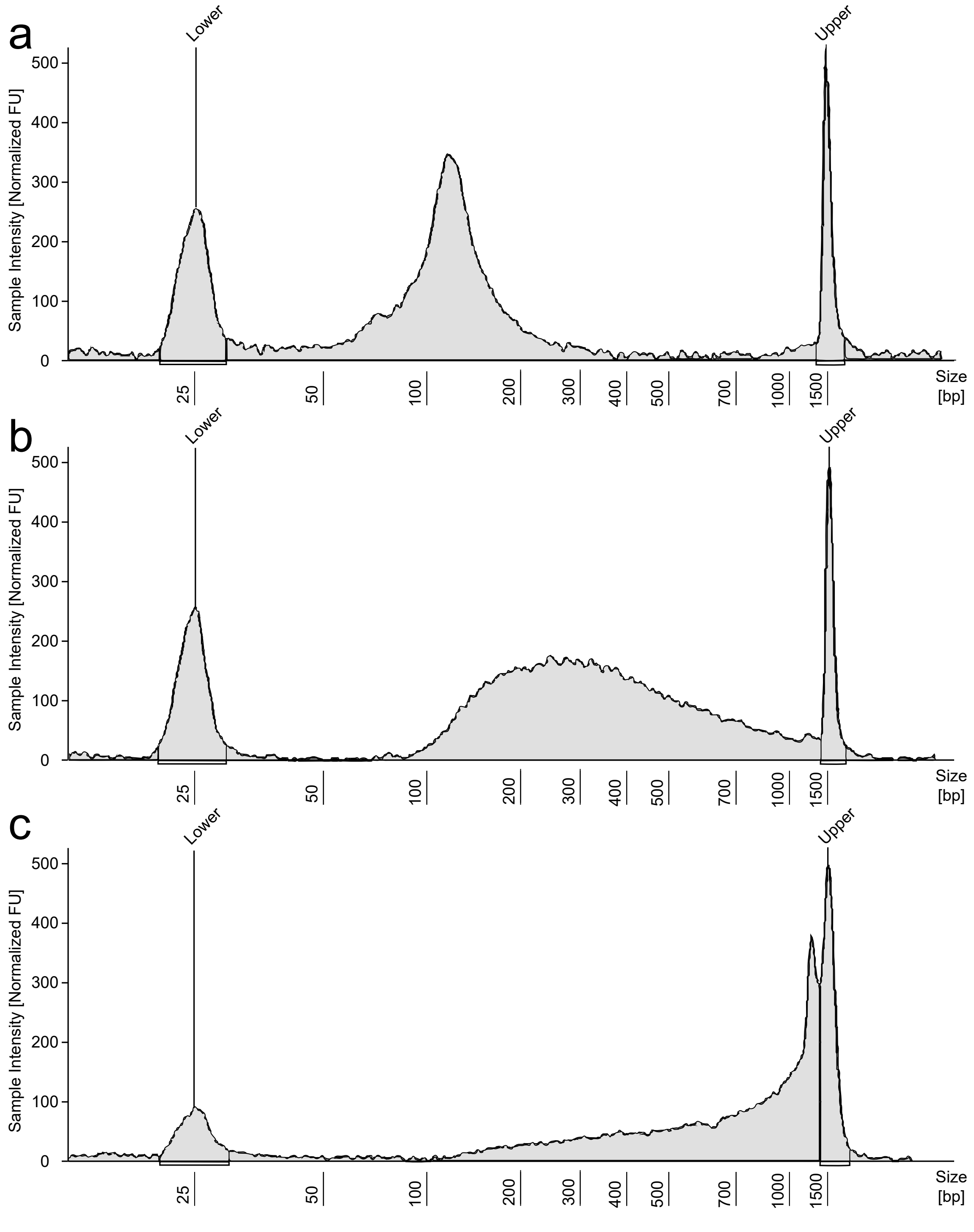

Supplementary Figure 3 The effect of $CaCl_2$ concentration on the size distribution of single-cell genomic DNA fragmented in droplets. MNase had a concentration of 0.01875 U/µl in droplets in these experiments. (a) 0.25 mM $CaCl_2$; (b) 0.1625 mM $CaCl_2$; (c) 0.0625 mM $CaCl_2$. The final sequencing libraries yielded under these conditions (having a volume of 20 µl) had concentrations of (a) 0.5 nM; (b) 2.5 nM; (c) 0.1 nM, measured by qPCR using the KAPA library quantification kit.

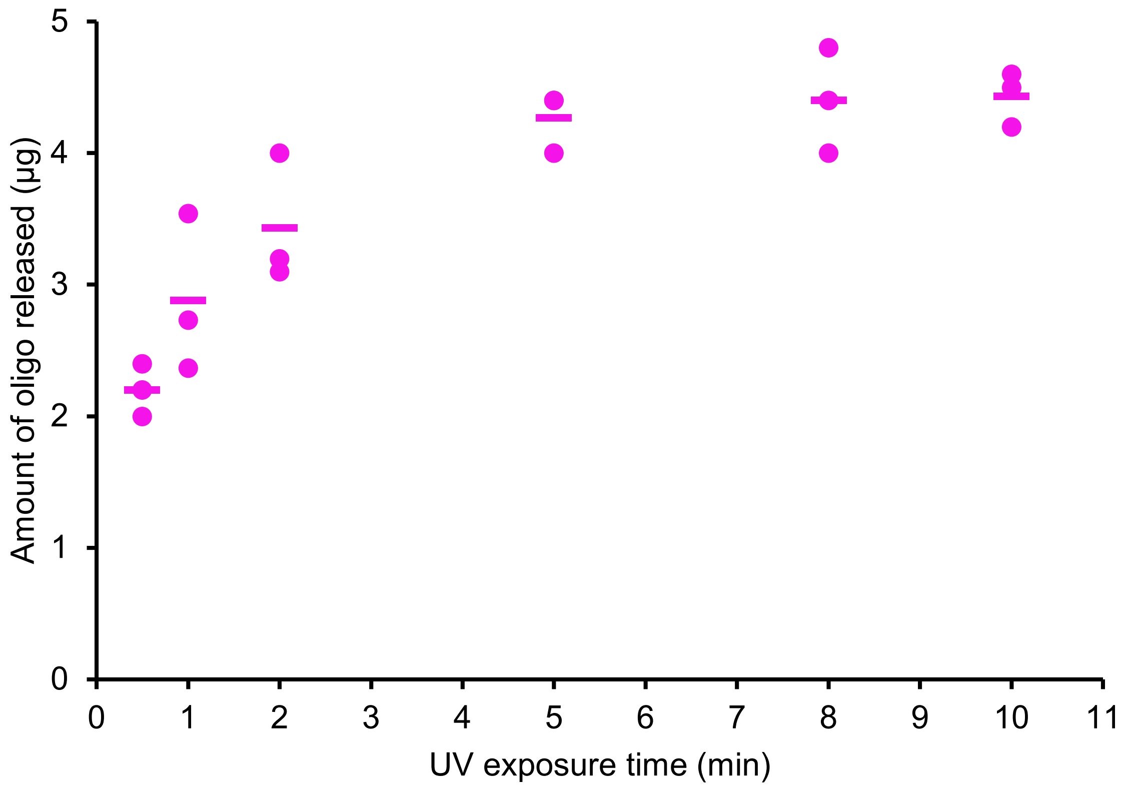

Supplementary Figure 4 The amount of oligonucleotide released from ~5,000 barcode beads after various UV exposure times. All experiments were conducted in triplicate, and the horizontal lines represent the mean. Source data are provided as a Source Data file.

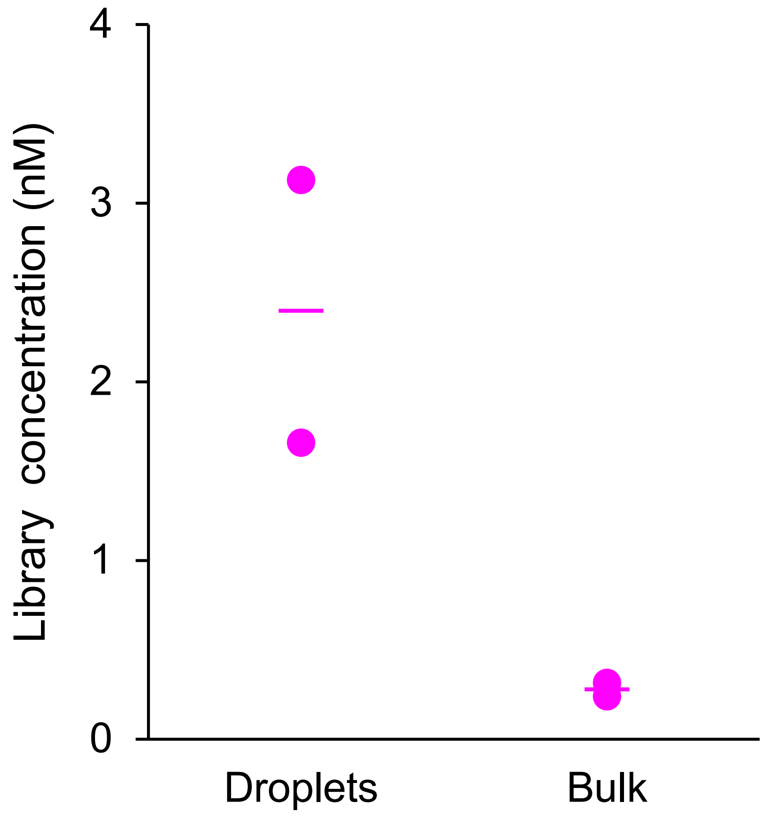

Supplementary Figure 5 Bisulfite conversion in droplets and bulk (in a tube). We followed Drop-BS protocol to construct these libraries (each starting with ~1,000 GM12878 single cells), except that the “bulk” ones had bisulfite conversion in a tube instead of in droplets. We measured the library concentration using a KAPA Library Quantification Kit (Roche, KK4824). All experiments were conducted in duplicate, and the horizontal lines represent the mean. Source data are provided as a Source Data file.

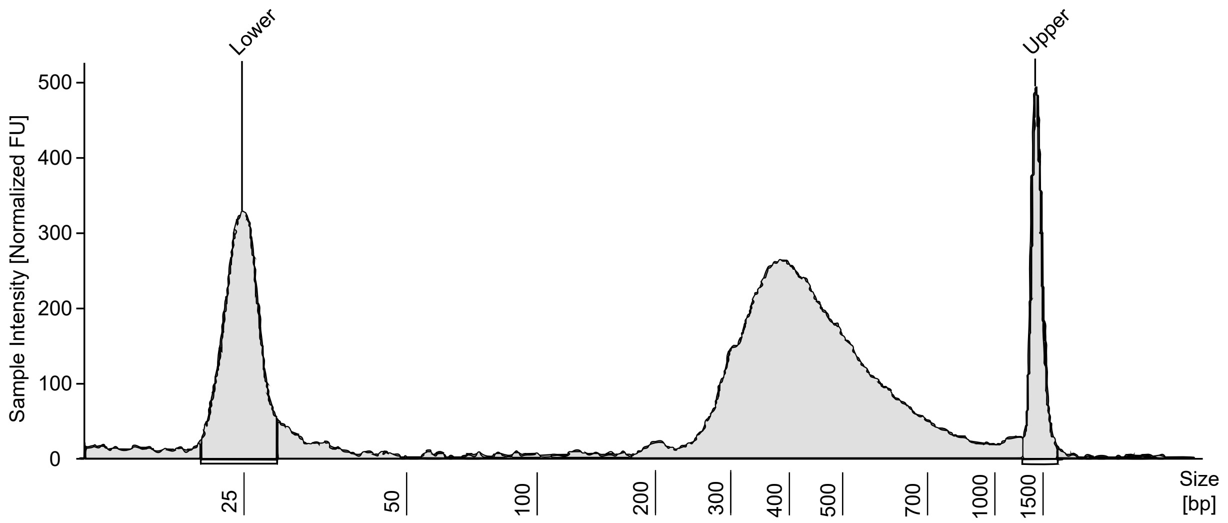

Supplementary Figure 6 The size profile of a Drop-BS sequencing library measured by an Agilent TapeStation.

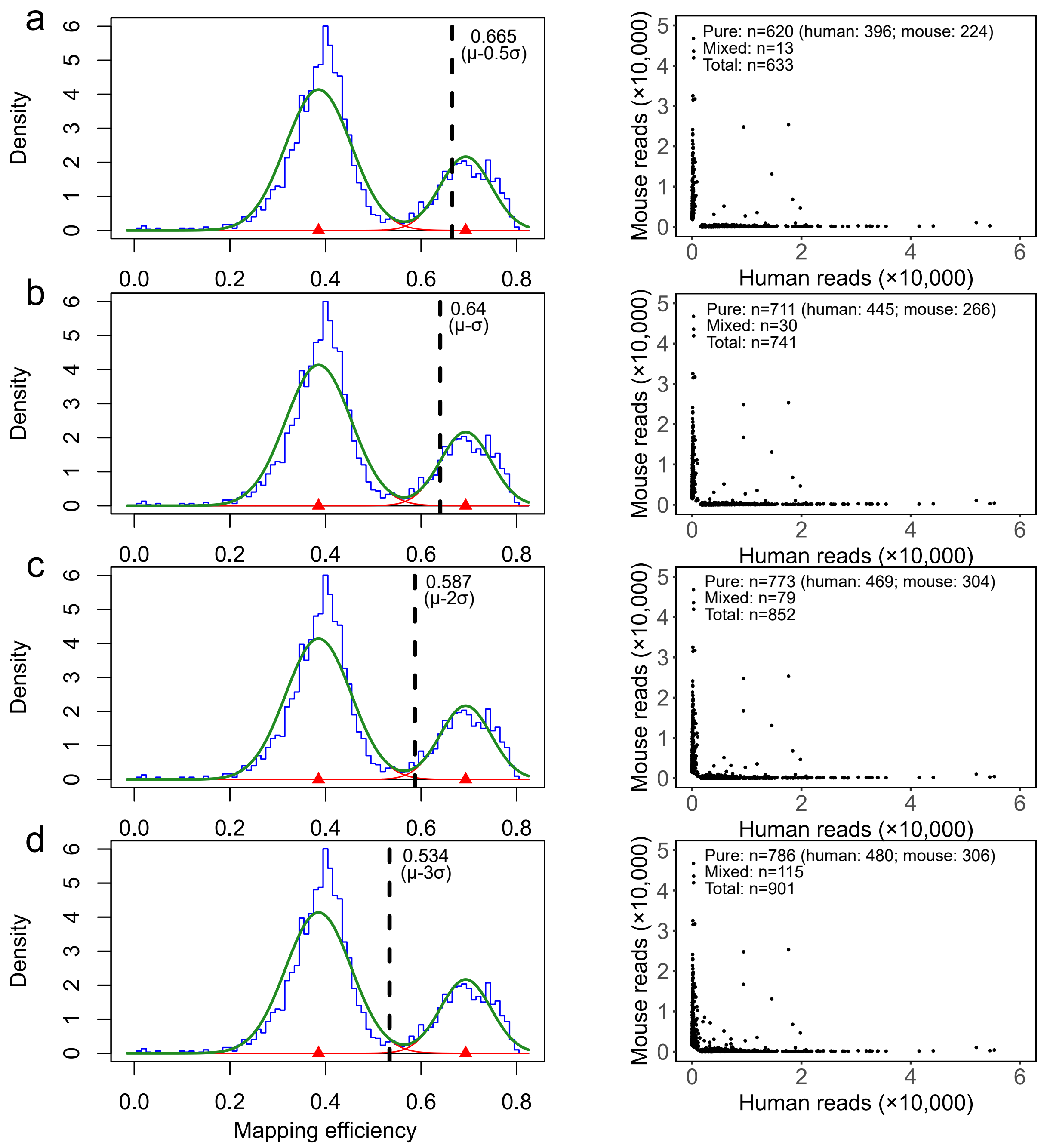

Supplementary Figure 7 Selection of cell-associated barcodes under various mapping efficiency cutoffs with human/mouse mixed cell sample. GM12878 and mouse brain nuclei were mixed at 1:1 ratio and Drop-BS library of ~1,000 cells were prepared. Each dot is a barcode bearing reads that can align to human genome or mouse genome. "Pure" barcodes refer to the ones with 90% or more of their reads aligned to one genome (human hg19 or mouse mm10). μ and σ are the mean and standard deviation of the fitted normal distribution on the right, respectively. (a) μ - 0.5σ; (b) μ - σ; (c) μ - 2σ; (d) μ - 3σ are used as cutoffs in the probability density plots (left) and the corresponding alignment of selected barcodes to the human and mouse genomes is shown (right). μ - σ is selected as the cutoff that balances the data quality (purity) and the number of cells covered. Source data are provided as a Source Data file.

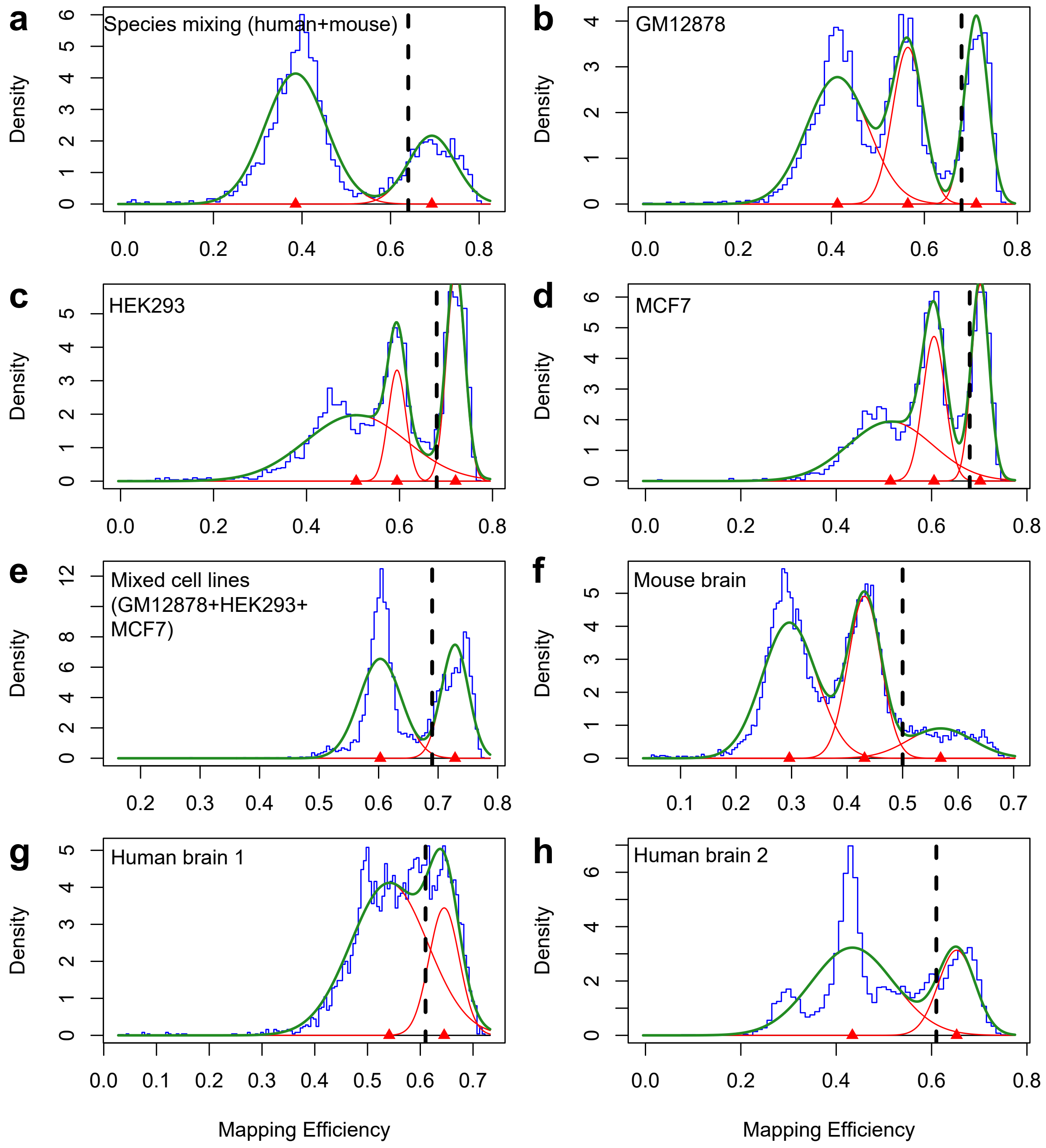

Supplementary Figure 8 Probability density plots of mapping efficiency for all samples and their corresponding cutoffs (μ-σ) for selection of cell-associated barcodes. (a) Mixed human and mouse cells; (b) GM12878 cells; (c) HEK293 cells; (d) MCF7 cells; (e) Mixed cell lines; (f) Mouse brain sample; (g) Human brain 1 sample; (h) Human brain 2 sample. Blue lines: density distributions of mapping efficiency of Drop-BS data. Red lines: fitted normal distributions. Green lines: combined fitted distributions. Black broken lines: the mapping efficiency cutoff, i.e. μ-σ of the normal distribution on the right. Source data are provided as a Source Data file.

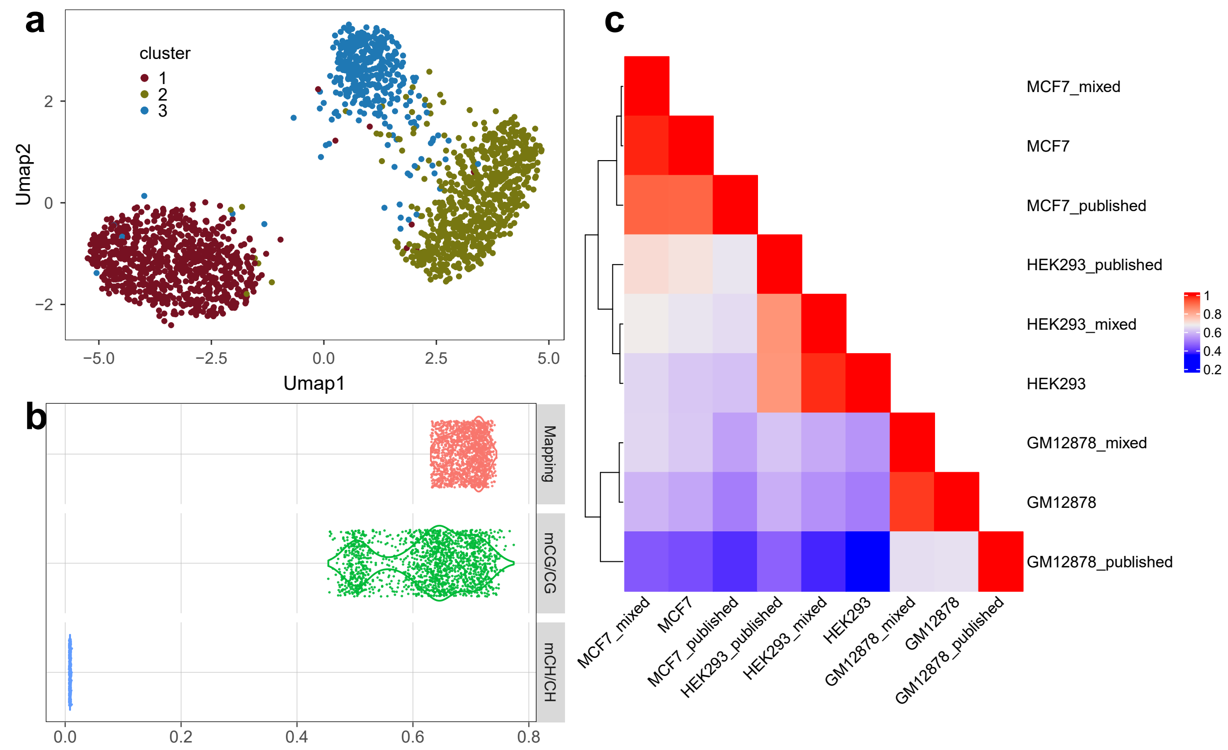

Supplementary Figure 9 Drop-BS data on cell lines (GM12878, HEK293, and MCF7) and their mixture (containing equal portions of the three cell lines). (a) UMAP visualization of Louvain clustering results on Drop-BS data of a mixture of three human cell lines (Cluster 1: MCF7; 2: HEK293; 3: GM12878). (b) CG methylation rate (mCG/CG), CH methylation rate (mCH/CH), and mapping efficiency of selected high-quality single-cell data from the mixed sample. (c) Pearson correlation and hierarchical clustering among the merged Drop-BS data and published methylomic data based on mCG/CG across equally spaced 1M genomic bins. “GM12878”, “HEK293”, and “MCF7” were the merged single-cell Drop-BS data when each cell line was individually profiled. “GM12878_mixed”, “HEK293_mixed”, “MCF7_mixed” were the merged single-cell Drop-BS data generated on each cell line by clustering the mixed sample. “GM12878_published”, “HEK293_published” and “MCF7_published” were published bulk methylomic data from ENCODE (ENCFF570TIL), GSM1254259, and GSM1328112, respectively. Source data are provided as a Source Data file.

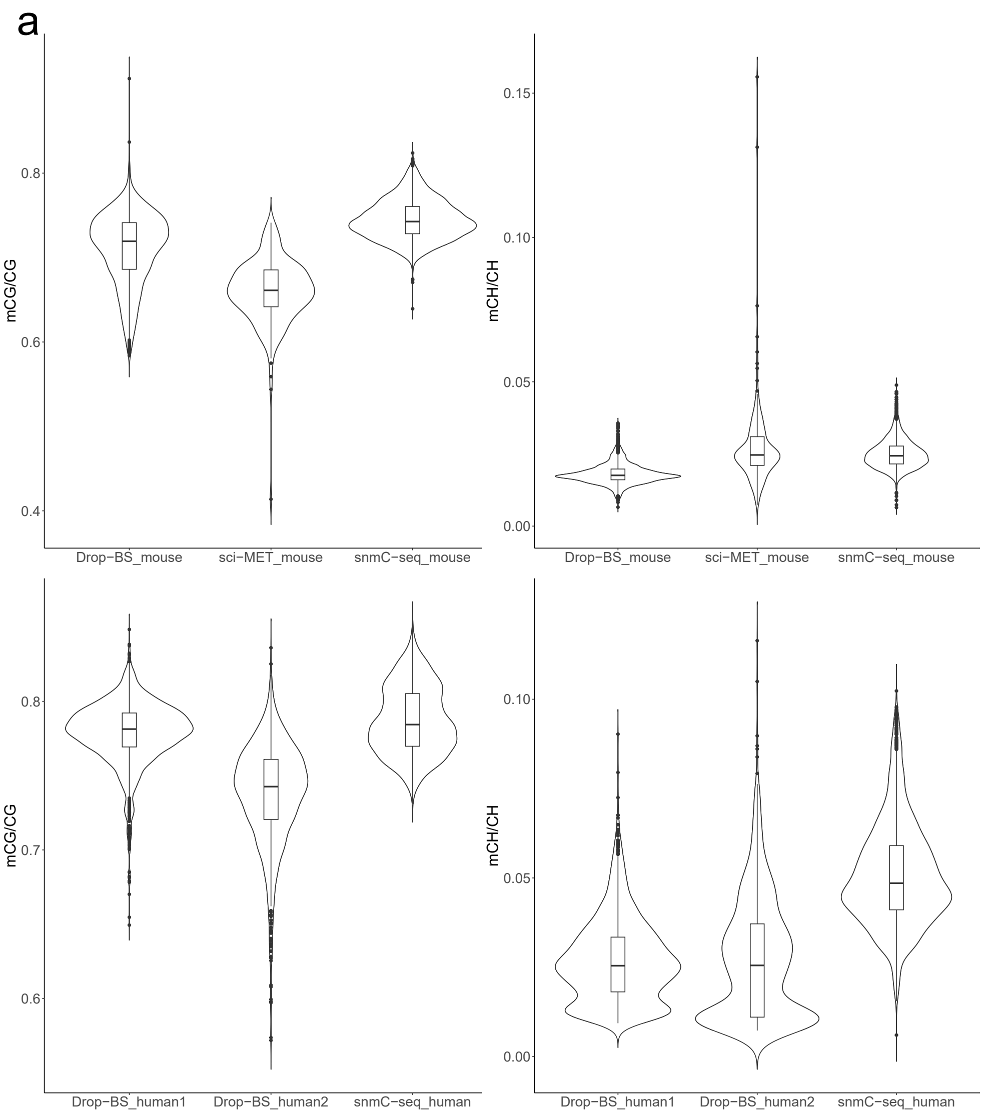

b

	DropBS mouse brain data	sci-MET mouse brain data	snmC-seq mouse brain data	DropBS human brain 1 data	DropBS human brain 2 data	snmC-seq human brain data
**mCG/CG**	71.73±3.77%	65.97±3.82%	74.49±2.34%	77.99±2.07%	73.80±3.39%	78.71±2.28%
**mCH/CH**	1.85±0.39%	2.73±1.50%	2.50±0.49%	2.70±1.16%	2.72±1.68%	5.09±1.44%

Supplementary Figure 10 (a) Violin plots and (b) Table of comparison of mCG/CG and mCH/CH of mouse and human brain samples profiled by Drop-BS and other techniques. Drop-BS data are on mouse (n=1123 cells) and human (n=1556 cells for human1 and n=1257 cells for human2) prefrontal cortex tissue. SnmC-seq profiled mouse (n=3377 cells) and human (n=2740 cells) frontal cortex neurons. sci-MET profiled mouse cortex tissue (n=210 cells). In the violin plots, the lower and upper hinges correspond to the first and third quartiles. The upper whisker extends from the hinge to the largest value no further than 1.5*IQR from the hinge. The lower whisker extends from the hinge to the smallest value at most 1.5*IQR of the hinge. Data beyond the end of the whiskers are plotted individually. Source data are provided as a Source Data file.

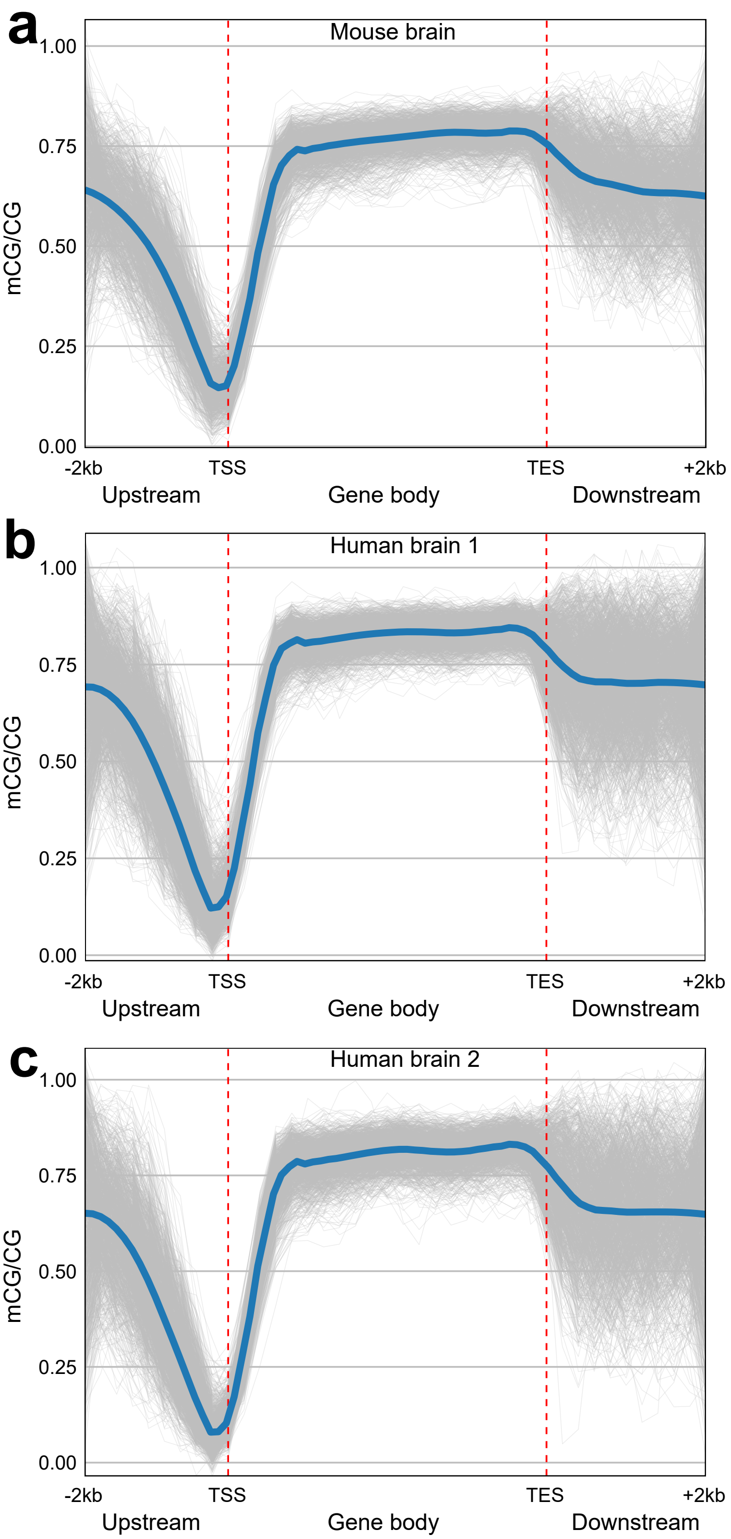

Supplementary Figure 11 Methylation level (mCG/CG) across upstream 2 kb of TSS (transcription start site), NCBI RefSeqGene body, and downstream 2 kb of TES (transcription termination site) for single cells (grey lines) and an average of all cells (blue line) for mouse PFC sample (a) and human PFC samples (b, c). Source data are provided as a Source Data file.

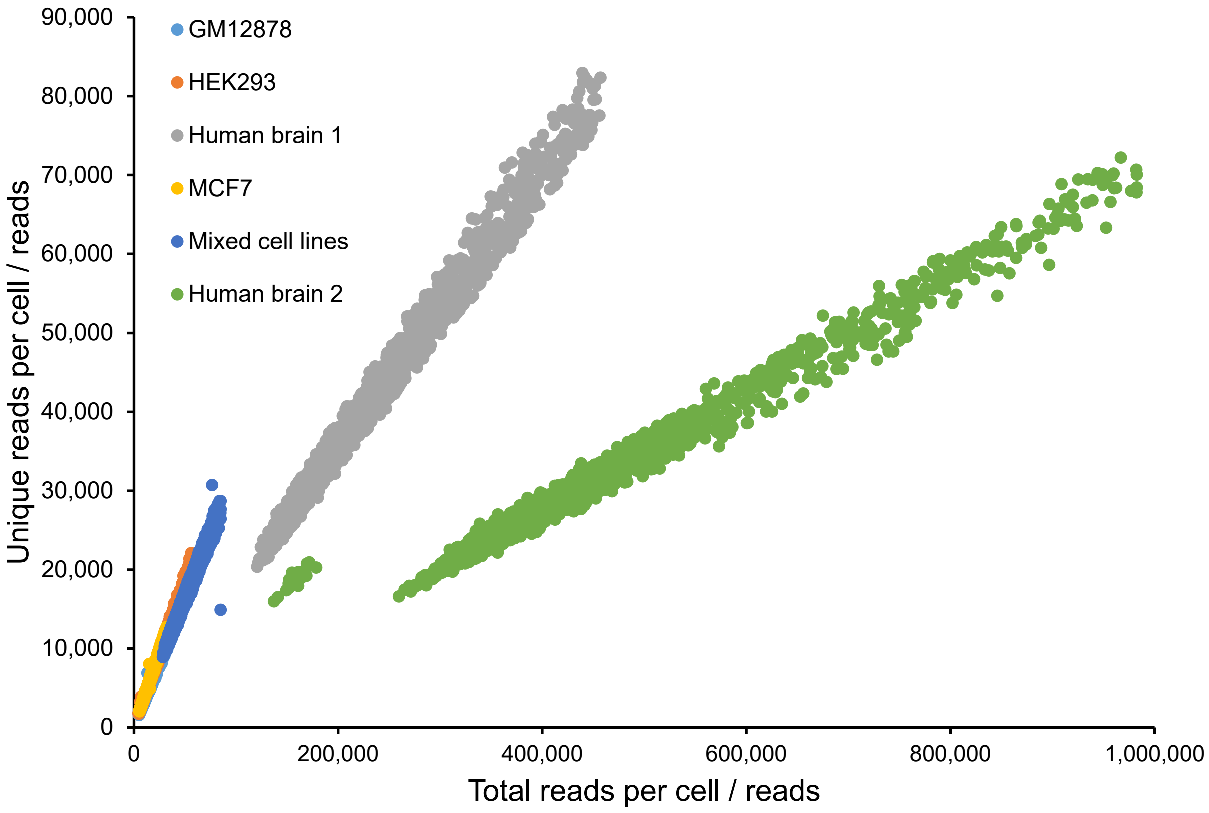

Supplementary Figure 12 The relationship between the total number of reads and the number of unique reads for each cell in Drop-BS datasets. Source data are provided as a Source Data file.
