# Supplementary material for: Droplet-based bisulfite sequencing for high-throughput profiling of single-cell DNA methylomes
Source: Nat Commun. 2023 Aug 3;14:4672. doi: 10.1038/s41467-023-40411-w (PMC10400590; doi:10.1038/s41467-023-40411-w)
Supplement: Supplementary file 2 — Description of Additional Supplementary Files Document [file 41467_2023_40411_MOESM2_ESM.pdf]

Description of Additional Supplementary Files Document

Supplementary Data

Supplementary Data 1. Metadata for Drop-BS data.

Supplementary Data 2. The average mCH/CH of each cluster in combined human brain 1+2 dataset on various functional elements.

Supplementary Data 3. DMRs and DMR-associated genes identified between excitatory and inhibitory neurons from human brains.

Supplementary Data 4. Comparison between the percentages of Drop-BS data (Human brain 1 dataset) on various functional elements and those of the genome occupied by the same elements including CpG Islands (UCSC annotation database), genic regions (NCBI RefSeq), promoter regions (2 kb regions upstream of transcription starting sites of NCBI RefSeq genes), repetitive regions (UCSC annotation database), and TFBS (UCSC annotation database).

Supplementary Data 5. Coverage of genomic regions by each cell in Drop-BS human brain 1 dataset.

Supplementary Data 6. Photomask for the droplet generation device.

Supplementary Data 7. Photomask for the bisulfite droplet device.

Supplementary Data 8. Photomask for the droplet fusion device.

Supplementary Movies

Supplementary Movie 1. Single nuclei encapsulation in the droplet generation device.

Supplementary Movie 2. Barcode droplet generation and scDNA droplet reinjection in the droplet fusion device.

Supplementary Movie 3. Barcode and scDNA droplets pairing in the droplet fusion device.

Supplementary Movie 4. Fusion of scDNA and barcode droplets under dielectrophoresis in the droplet fusion device. The alternating current field was applied via the salt channel electrodes on both sides of the droplet channel.

Supplementary Code

Codes for data analysis of Drop-BS data.
