# Supplementary figures and images for: Droplet-based bisulfite sequencing for high-throughput profiling of single-cell DNA methylomes
Source: Nat Commun. 2023 Aug 3;14:4672. doi: 10.1038/s41467-023-40411-w (PMC10400590; doi:10.1038/s41467-023-40411-w)

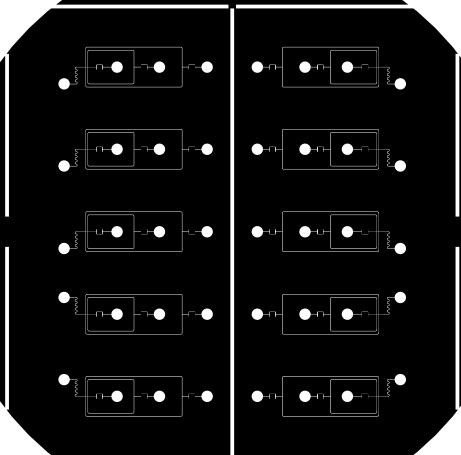

Supplement: Supplementary file 8 — Supplementary Data 6 [file 41467_2023_40411_MOESM8_ESM.pdf]

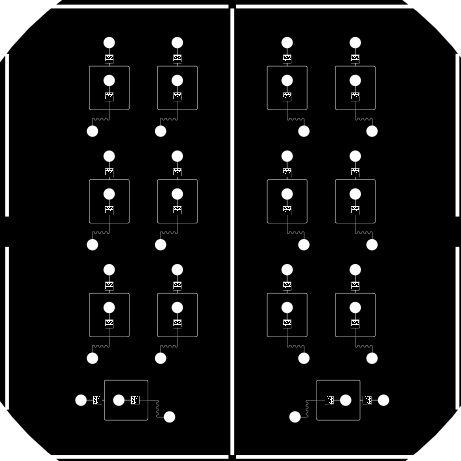

5000  $\mu\text{m}$

Supplement: Supplementary file 9 — Supplementary Data 7 [file 41467_2023_40411_MOESM9_ESM.pdf]

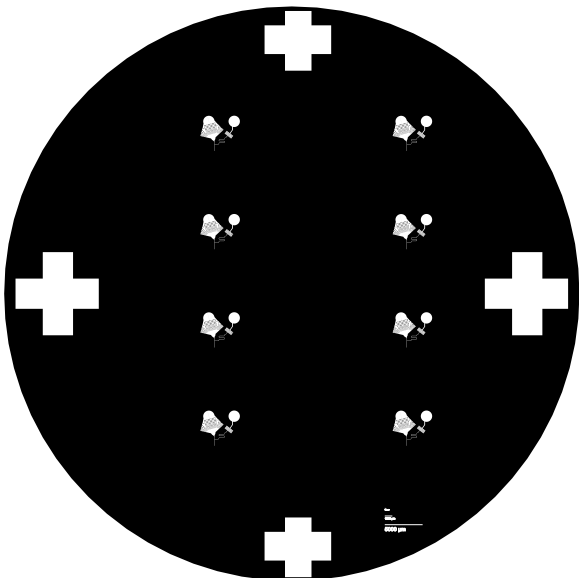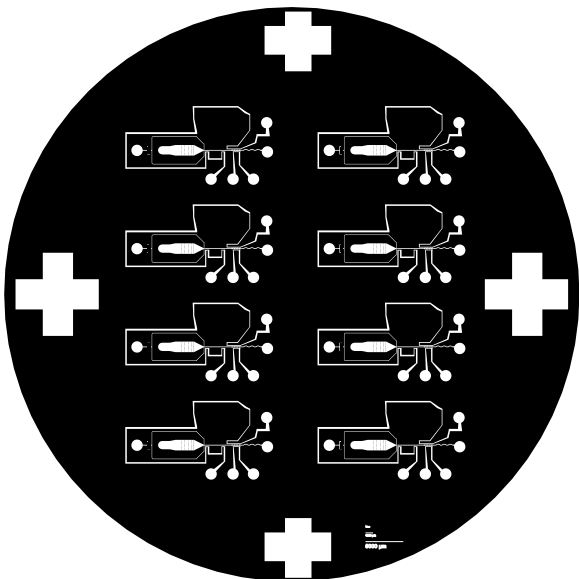

Supplement: Supplementary file 10 — Supplementary Data 8 [file 41467_2023_40411_MOESM10_ESM.pdf]
