# Supplementary material for: Droplet-based bisulfite sequencing for high-throughput profiling of single-cell DNA methylomes
Source: Nat Commun. 2023 Aug 3;14:4672. doi: 10.1038/s41467-023-40411-w (PMC10400590; doi:10.1038/s41467-023-40411-w)
Supplement: Supplementary file 16 — Reporting Summary [file 41467_2023_40411_MOESM16_ESM.pdf]

nature portfolio

Corresponding author(s): Chang Lu

Last updated by author(s): Jul 4, 2023



# Reporting Summary

Nature Portfolio wishes to improve the reproducibility of the work that we publish. This form provides structure for consistency and transparency in reporting. For further information on Nature Portfolio policies, see our Editorial Policies and the Editorial Policy Checklist.

## Statistics

For all statistical analyses, confirm that the following items are present in the figure legend, table legend, main text, or Methods section.

| n/a | Confirmed | |
|---|---|---|
| ☐ | ☒ | The exact sample size (*n*) for each experimental group/condition, given as a discrete number and unit of measurement |
| ☐ | ☒ | A statement on whether measurements were taken from distinct samples or whether the same sample was measured repeatedly |
| ☐ | ☒ | The statistical test(s) used AND whether they are one- or two-sided<br>*Only common tests should be described solely by name; describe more complex techniques in the Methods section.* |
| ☐ | ☒ | A description of all covariates tested |
| ☐ | ☒ | A description of any assumptions or corrections, such as tests of normality and adjustment for multiple comparisons |
| ☐ | ☒ | A full description of the statistical parameters including central tendency (e.g. means) or other basic estimates (e.g. regression coefficient) AND variation (e.g. standard deviation) or associated estimates of uncertainty (e.g. confidence intervals) |
| ☐ | ☒ | For null hypothesis testing, the test statistic (e.g. *F*, *t*, *r*) with confidence intervals, effect sizes, degrees of freedom and *P* value noted<br>*Give P values as exact values whenever suitable.* |
| ☒ | ☐ | For Bayesian analysis, information on the choice of priors and Markov chain Monte Carlo settings |
| ☐ | ☒ | For hierarchical and complex designs, identification of the appropriate level for tests and full reporting of outcomes |
| ☐ | ☒ | Estimates of effect sizes (e.g. Cohen's *d*, Pearson's *r*), indicating how they were calculated |

*Our web collection on statistics for biologists contains articles on many of the points above.*

## Software and code

Policy information about availability of computer code

| | |
|---|---|
| Data collection | Drop-BS libraries were sequenced on a Novaseq 6000 system with standard sequencing protocol. |
| Data analysis | umi_tools (v1.0.1), Tim Galore! (v0.6.7), idemp(v1), Bismark (v0.23.0), bowtie2 (v2.3.5.1), Picard (v2.14.0), methylKit (v1.10.0), R (v 4.1.2), irlba package (v 2.3.5), igraph package (v1.3.1), UMAP (v 0.2.8.0), pheatmap (v1.0.12). |

For manuscripts utilizing custom algorithms or software that are central to the research but not yet described in published literature, software must be made available to editors and reviewers. We strongly encourage code deposition in a community repository (e.g. GitHub). See the Nature Portfolio guidelines for submitting code & software for further information.

## Data

Policy information about availability of data

All manuscripts must include a data availability statement. This statement should provide the following information, where applicable:

- Accession codes, unique identifiers, or web links for publicly available datasets
- A description of any restrictions on data availability
- For clinical datasets or third party data, please ensure that the statement adheres to our policy

The Drop-BS data on cell lines and mouse brain and the processed Drop-BS data on human brain have been deposited in Gene Expression Omnibus (GEO) database under accession number GSE204691 [https://www.ncbi.nlm.nih.gov/geo/query/acc.cgi?acc=GSE204691]. The raw Drop-BS data on postmortem human brains were deposited in dbGaP under accession number phs002123.v2.p1 [https://www.ncbi.nlm.nih.gov/projects/gap/cgi-bin/study.cgi?study_id=phs002123.v2.p1] and can

be obtained via authorized access from the database. sci-MET datasets from GEO, accession GSE112554 [https://www.ncbi.nlm.nih.gov/geo/query/acc.cgi?acc=GSE112554]. snmC-seq datasets from GEO, accsssion GSE97179 [https://www.ncbi.nlm.nih.gov/geo/query/acc.cgi?acc=GSE97179]. GM12878 whole-genome bisulfite sequencing (WGBS) data from ENCODE, accession ENCSR890UQO/ENCFF570TIL [https://www.encodeproject.org/files/ENCFF570TIL/]. HEK293 WGBS data from GEO, accession GSM1254259 [https://www.ncbi.nlm.nih.gov/geo/query/acc.cgi?acc=GSM1254259]. MCF7 WGBS data from GEO, accession GSM1328112 [https://www.ncbi.nlm.nih.gov/geo/query/acc.cgi?acc=GSM1328112]. CpG Islands from UCSC [https://genome.ucsc.edu/cgi-bin/hgTables?hgsid=1650303542_VV4O0tu8uPaMfkCNaMWaFPsrzRaY&clade=mammal&org=Human&db=hg19&hgta_group=allTracks&hgta_track=cpgIslandExt&hgta_table=0&hgta_regionType=genome&position=chr2%3A25%2C383%2C722-25%2C391%2C559&hgta_outputType=primaryTable&hgta_outFileName=CpG Islands.bed]. Genic regions from UCSC [https://genome.ucsc.edu/cgi-bin/hgTables?hgsid=1650303542_VV4O0tu8uPaMfkCNaMWaFPsrzRaY&clade=mammal&org=Human&db=hg19&hgta_group=genes&hgta_track=refSeqComposite&hgta_table=0&hgta_regionType=genome&position=chr2%3A25%2C383%2C722-25%2C391%2C559&hgta_outputType=primaryTable&hgta_outFileName=]. Repetitive regions from UCSC [https://genome.ucsc.edu/cgi-bin/hgTables?hgsid=1650303542_VV4O0tu8uPaMfkCNaMWaFPsrzRaY&clade=mammal&org=Human&db=hg19&hgta_group=rep&hgta_track=refSeqComposite&hgta_table=0&hgta_regionType=genome&position=chr2%3A25%2C383%2C722-25%2C391%2C559&hgta_outputType=primaryTable&hgta_outFileName=]. TFBS from UCSC [https://genome.ucsc.edu/cgi-bin/hgTables?hgsid=1650303542_VV4O0tu8uPaMfkCNaMWaFPsrzRaY&clade=mammal&org=Human&db=hg19&hgta_group=allTracks&hgta_track=encRegTfbsClustered&hgta_table=0&hgta_regionType=genome&position=chr2%3A25%2C383%2C722-25%2C391%2C559&hgta_outputType=primaryTable&hgta_outFileName=]. Source data are provided with this paper.

## Human research participants

Policy information about studies involving human research participants and Sex and Gender in Research.

| | |
|---|---|
| Reporting on sex and gender | The research involves only postmortem human brain samples, thus does not involve human research participants. |
| Population characteristics | The research involves only postmortem human brain samples, thus does not involve human research participants. |
| Recruitment | The research involves only postmortem human brain samples, thus does not involve human research participants. |
| Ethics oversight | The research involves only postmortem human brain samples, thus does not involve human research participants. |

Note that full information on the approval of the study protocol must also be provided in the manuscript.

# Field-specific reporting

Please select the one below that is the best fit for your research. If you are not sure, read the appropriate sections before making your selection.

☒ Life sciences ☐ Behavioural & social sciences ☐ Ecological, evolutionary & environmental sciences

For a reference copy of the document with all sections, see nature.com/documents/nr-reporting-summary-flat.pdf

# Life sciences study design

All studies must disclose on these points even when the disclosure is negative.

| | |
|---|---|
| Sample size | >5741 single cells from cell lines and >3936 single cells from brains were sequenced. Our sample size is comparable to studies that validate competing technologies. |
| Data exclusions | We used certain criteria to remove low-quality barcodes. Details were described in the Method section. No data exclusion otherwise. |
| Replication | Drop-BS experiments were performed across a total of >9677 single cells. All attempts at replication were successful |
| Randomization | No randomization was used in the experiments. Our study is on technology development and validation. Randomization is not relevant. |
| Blinding | Investigators were not blinded during data collection and analysis. Our study is on technology development and validation. Blinding is not relevant. |

# Reporting for specific materials, systems and methods

We require information from authors about some types of materials, experimental systems and methods used in many studies. Here, indicate whether each material, system or method listed is relevant to your study. If you are not sure if a list item applies to your research, read the appropriate section before selecting a response.

## Materials & experimental systems

| n/a | Involved in the study | |
|---|---|---|
| ☒ | ☐ | Antibodies |
| ☐ | ☒ | Eukaryotic cell lines |
| ☒ | ☐ | Palaeontology and archaeology |
| ☐ | ☒ | Animals and other organisms |
| ☒ | ☐ | Clinical data |
| ☒ | ☐ | Dual use research of concern |

## Methods

| n/a | Involved in the study | |
|---|---|---|
| ☒ | ☐ | ChIP-seq |
| ☒ | ☐ | Flow cytometry |
| ☒ | ☐ | MRI-based neuroimaging |

# Eukaryotic cell lines

Policy information about cell lines and Sex and Gender in Research

| | |
|---|---|
| Cell line source(s) | GM12878: a lymphoblastoid cell line produced from the blood of a female donor, purchased from Coriel Institute for Medical Research (#CEPH/UTAH. Pedigree 1463). HEK293: a kidney epithelial cell line (gender: female), purchased from ATCC (CRL-1573). MCF7: a human breast cancer cell line (gender: female), purchased from ATCC (HTB-22). |
| Authentication | All cell lines were purchased commercially and authenticated by the vendors. No additional authentication was conducted. |
| Mycoplasma contamination | Negative (routinely tested). |
| Commonly misidentified lines (See ICLAC register) | No commonly misidentified cell lines were used in the study. |

# Animals and other research organisms

Policy information about studies involving animals; ARRIVE guidelines recommended for reporting animal research, and Sex and Gender in Research

| | |
|---|---|
| Laboratory animals | C57BL/6J mice were purchased from Jackson Laboratory and housed in standard breeding cages at constant temperature (22±1°C) and relative humidity (50%) with 12-h light/12-h dark cycles and food and water ad libitum. 10-week-old male mice were sacrificed by compressed CO2 followed by cervical dislocation. |
| Wild animals | No wild animals were used in the study. |
| Reporting on sex | Sex is not relevant in this research. The sex of the mice used (male) is reported. |
| Field-collected samples | No field collected samples were used in the study. |
| Ethics oversight | This study was approved by the Institutional Animal Care and Use Committee (IACUC) at Virginia Tech. |

Note that full information on the approval of the study protocol must also be provided in the manuscript.
